# Cerebral blood flow regulation is not acutely altered after a typical number of headers in women footballers

**DOI:** 10.3389/fneur.2022.1021536

**Published:** 2022-11-21

**Authors:** Jacob Jack, Alex Woodgates, Oliver Smail, Felix Brown, Katie Lynam, Alice Lester, Genevieve Williams, Bert Bond

**Affiliations:** Exeter Head Impacts, Brain Injury and Trauma (ExHIBIT) Research Group, Sport and Health Sciences, University of Exeter, Exeter, United Kingdom

**Keywords:** soccer, cerebrovascular reactivity, carbon dioxide, autoregulation, neurovascular coupling

## Abstract

**Background:**

The repeated act of heading has been implicated in the link between football participation and risk of neurodegenerative disease, and acutely alters cerebrovascular outcomes in men. This study assessed whether exposure to a realistic number of headers acutely influences indices of cerebral blood flow regulation in female footballers.

**Methods:**

Nineteen female players completed a heading trial and seated control trial on two separate days. The heading trial involved six headers in 1 h (one every 10 min), with the ball traveling at 40 ± 5 km/h. Cerebrovascular reactivity to hypercapnia and hypocapnia was determined using serial breath holding and hyperventilation attempts. Dynamic cerebral autoregulation (dCA) was assessed by scrutinizing the relationship between cerebral blood flow and mean arterial blood pressure during 5 min of squat stand maneuvers at 0.05 Hz. Neurovascular coupling (NVC) was quantified as the posterior cerebral artery blood velocity response to a visual search task. These outcomes were assessed before and 1 h after the heading or control trial.

**Results:**

No significant time by trial interaction was present for the hypercapnic (*P* = 0.48, $\eta_{p}^{2}$ = 0.05) and hypocapnic (*P* = 0.47, $\eta_{p}^{2}$ = 0.06) challenge. Similarly, no significant interaction effect was present for any metric of dCA (*P* > 0.12, $\eta_{p}^{2}$ < 0.16 for all) or NVC (*P* > 0.14, $\eta_{p}^{2}$ < 0.15 for all).

**Conclusion:**

The cerebral blood flow response to changes in carbon dioxide, blood pressure and a visual search task were not altered following six headers in female footballers. Further study is needed to observe whether changes are apparent after more prolonged exposure.

## Introduction

Recent evidence highlights that former professional male footballers (soccer) are at an increased risk of neurodegenerative disease (1). A follow up publication by the same research group highlighted that this risk differed by playing position (2). Whilst the authors were not able to determine the position-specific exposure which might explain this finding, the repeated act of heading—which is unique to football—might explain at least part of this relationship (3–5).

Footballers with no history of concussion have been shown to have widespread differences in cerebral white matter integrity (6) and impaired cerebrovascular reactivity to carbon dioxide (CO_2_) (7) compared to those who participate in non-contact sports. Recent interventional studies have also demonstrated that heading acutely alters the regulation of cerebral blood flow in response to oscillations in blood pressure [dynamic cerebral autoregulation, dCA (8)] and a visual search task [neurovascular coupling, NVC (9)]. The regulation of brain blood flow to these stimuli is critical, and alterations in the ability to do so are implicated in the progression of neurodegenerative disease (10–12). It is thought that the repeated exposure to these transient alterations in cerebrovascular regulation post heading might explain part of the increased risk of neurodegenerative disease associated with a playing career. Therefore, there is value in understanding whether the exposure to the number of headers typical of a match or training session acutely influences these indices of cerebrovascular regulation.

A current limitation of the extant evidence base, is that an unrealistic heading stimulus (i.e., 40 headers in 20 min) has been used (8, 9). In comparison, the average number of headers performed by women within a 90 min match or training session is three (13–15), with the typical maximum reported to be seven (16, 17). Whilst such studies have value in furthering our understanding of the role heading might play regarding the risk of future neurodegenerative disease, they are in danger of exaggerating any relationship. However, acute alterations in intracranial pressure (18) and postural control (19–21) have been observed following just six and 10–12 headers, respectively. Whether the exposure a heading stimulus consistent with training or match play acutely alters indices of cerebral blood flow regulation is yet unknown.

A further limitation of the evidence linking football participation (and, by implication, heading) with an increased risk of neurodegenerative disease, is that it is solely driven by data in male former footballers (1, 2). No equivalent studies are available for women, largely because no such cohort evidence base currently exists. However, initial evidence indicates that women may experience greater head accelerations during heading than men (22, 23), which might explain the greater magnitude of white matter alterations in female footballers even when matched for heading exposure (24). This potential discrepancy between the sexes is a considerable concern. The effect of heading on indices of cerebral blood flow regulation in women is currently poorly understood, but it has been demonstrated that the accumulated exposure to headers over a season is associated with a blunted cerebrovascular response to the hypercapnic challenge of breath holding in adolescent women footballers (25). Given the current efforts to continue increasing the number of women playing football (26), the purpose of this study was to identify whether heading acutely influences cerebrovascular reactivity to CO_2_, dCA, and NVC in female footballers.

## Materials and methods

### Participants

Ethical approval was obtained from the University of Exeter Sport and Health Sciences Ethics Committee (2012-A-01) and participants provided written informed consent prior to any data collection. The study was conducted in accordance with 1964 Declaration of Helsinki. Nineteen female footballers (10 defenders, 5 midfielders, 4 attackers) were recruited from the University football club (Table 1). Exclusion criteria included any contraindications to performing headers, including a history of migraines or seizures, the incidence of concussion within a 2 months period before visiting the laboratory, and not being an outfield player.

**Table 1:** Participant characteristics.

	**Mean ± Standard deviation**
Age (y)	21.9 ± 3.1
Stature (m)	1.64 ± 0.05
Mass (kg)	64.3 ± 7.9
Body mass index (kg/m^2^)	24.0 ± 2.9
Playing experience (y)	11.7 ± 5.4

Data are presented as the mean ± standard deviation. *N* = 19.

### Study design

Participants were accustomed to all procedures prior to data collection. Thereafter, participants reported to the laboratory on two further occasions to complete the control or heading intervention, which were performed in a counterbalanced order and separated by at least 7 days. Parameters of interest (outlined below) were measured before and 1 h after the control (time-matched seated rest) or heading protocol. These outcomes were always determined in the same order. To best reflect the typical exposure to headers in training or a match (14, 16, 17), participants were required to complete a total of six headers in an hour—one every 10 min. A motorized ball launcher (Ball Launcher Pro Trainer), positioned 15 m from the participants, was used to standardize the ball speed (40 ± 5 km/h) and trajectory, in line with other investigations (21, 23, 27). Participants were instructed to purposefully header the ball toward a researcher, perpendicular to the flight of the ball. In this way, we intended to replicate a cross into the box, which might cause an attacker to the head the ball toward goal, or a defender to head the ball to safety. The ball was the official size 5 UEFA Women's Champions League football, and always pressurized to the regulation 12 psi.

### Instrumentation

Cerebral artery blood velocity was quantified using transcranial Doppler sonography (Multidop, DWL), in line with contemporary guidelines (28), for the assessment of reactivity to CO_2_, CA, and NVC. Briefly, blood velocity in the right middle cerebral artery (MCAv; for CO_2_ reactivity and dCA) and posterior cerebral artery (PCAv; for NVC) was quantified using a 2 MHz probe over the transtemporal acoustic window, which was held in place using a customisable headset (DiaMon, DWL). The cerebral artery was identified *via* assessment of velocity and waveform, confirmed either by carotid compression or opening and closing of the eyes, and optimized by adjusting the signal depth (29). The position of the probe and the depth of the scan were noted, and efforts were made to replicate these within and between days for each participant. Beat-by-beat mean arterial pressure (MAP) was continuously measured by finger photoplethysmography (Finometer PRO, Netherlands). Breath by breath end tidal partial pressures of carbon dioxide (P_ET_CO_2_) were measured (ML206 Gas Analyzer, ADInstruments) as a surrogate of arterial carbon dioxide partial pressure (30) in order to confirm the timing of each breath hold and hyperventilation attempt. Finally, a three-lead electrocardiogram (ECG) was worn for R-R interval calibration of the MCAv and MAP data during the assessment of dCA. MCAv or PCAv, MAP, P_ET_ CO_2_ and the ECG signal were sampled continuously at 200 Hz using an analog-to-digital converter (Powerlab; model-−8/30, ADInstruments, Colorado Springs, CO, United States) linked with a laptop computer for subsequent integration (Lab Chart version 8, ADInstruments).

### Assessment of cerebrovascular reactivity to CO_2_

Cerebrovascular reactivity to hypercapnia and hypocapnia was inferred using a repeated breath holding and separate hyperventilation protocol, respectively. Such an approach is advocated as an alternative to inhalation of higher concentrations of CO_2_ (31–33), and we replicated a breath holding and hyperventilation protocol which is sensitive to alterations post concussion (34). Participants were coached to perform both techniques prior to any data collection, with particular instruction to avoid the Valsalva maneuver during breath holding. Following acclimation to the laboratory, baseline MCAv, MAP, and P_ET_CO_2_ data were collected for 1 min. Then, after a normal inspiration, subjects completed a 20 s breath hold. The researcher provided a countdown for each subject for the breath hold. Each attempt was held for 20 s and was followed by 40 s of recovery (normal breathing). This protocol of breath hold-recovery was repeated five times (34). The change in P_ET_CO_2_ from baseline to the first expiration post breath hold was calculated in order to infer alterations in arterial CO_2_ concentration.

Following recovery from the breath holding protocol, participants completed a new 1 min baseline of normal breathing before hyperventilating at 36 breaths per min for 20 s, followed by 40 s recovery (unpaced breathing). This was repeated a further 4 times (34). Cadence during the hyperventilation protocol was provided by an electronic metronome and supervised by a researcher. P_ET_CO_2_ was measured throughout. Cerebrovascular reactivity was quantified as the percentage change in MCAv immediately after each breath hold and hyperventilation attempt (34). The absolute change in MCAv per unit change in P_ET_CO_2_ following each breath hold and hyperventilation attempt was also scrutinized in order to account for any differences in P_ET_CO_2_ (35).

### Assessment of dCA

Dynamic CA was assessed once it was confirmed that participants had returned to a physiological steady following the hyperventilation challenge. Indices of dCA were assessed by scrutinizing the relationship between MAP and MCAv during 5 min of repeatedly transitioning from a ~ 90 degree squat position to standing at a frequency of 0.05 Hz, in accordance with contemporary recommendations (36). This approach provides large oscillations in MAP, ensures excellent linearity between input (MAP) and output (MCAv) signals, and superior within and between day reliability for dCA metrics (36, 37). Throughout the squat-stand procedures participants were asked to maintain the Finapres at heart-height, in order to abate any movement artifact and minimize hydrostatic errors in the blood-pressure data (38). Due to finger photoplethysmography equipment failure in 4 trials, data are presented for 15 participants only.

Coherence, gain, normalized gain (percentage change in MCAv per unit of MAP; %.mmHg^−1^) and phase of the actively driven blood-pressure oscillations were generated using transfer function analysis by extracting at the point estimate of the selected driven frequency (0.05 Hz), in accordance with current guidelines (36, 39) and similar investigations in this field (8, 40, 41), using dedicated software (Elucimed, Ensemble-R).

### Assessment of NVC

Upon completion of the squat-stand maneuvers for dCA, participants sat and rested whilst the researchers insonated the PCA. This typically ensured >10 min of rest, however PCAv and MAP were visually checked to confirm a steady state, resting response prior to the start of the NVC protocol. NVC was quantified using the visual search task approach advocated in recent methodological guidelines (42) and in line with other investigations in this field (9). Following a 1 min resting baseline (with eyes closed), participants were instructed to open their eyes and perform a visual search task (“Where's Wally”^®^) for 40 s, followed by a 20 s eyes closed recovery. If Wally was found during this time, participants were provided with a new picture in which they had to find the character. Thus, each visual search challenge lasted the full 40 s, and this approach is understood to be highly engaging compared to other visual stimuli (43). This process was repeated 5 times in total, which has been shown to provide excellent reproducibility (44).

The PCAv and MAP response to each 40 s eyes open transition was scrutinized prior to ensemble averaging the 5 attempts. In line with others (9, 42, 44, 45), data are presented as the averaged, absolute and percentage increase in PCAv in the first 30 s of each search attempt above the last 5 sec of averaged eyes closed baseline. The time to peak PCAv was noted, and the total hyperaemic response was calculated as the area under the PCAv curve vs. time, adjusted to the eyes closed baseline. Four participants were removed from analysis due to poor PCAv signal quality.

### Statistical analyses

All data are presented as mean ± standard deviation. Statistical analyses were completed using SPSS, version 26 (IBM), with statistical significance set *a priori* at *P* < 0.05. The assumptions of sphericity and normality were checked using Mauchly's and the Shapiro-Wilk tests, respectively. The cerebrovascular responses to the heading and control protocol were explored using a series of separate, two-way repeated measures ANOVA tests. Specifically, resting hemodynamics, dCA metrics and NVC outcomes were scrutinized for a trial by time interaction effect, whilst the response to breath holding and hyperventilation were assessed using a time (pre vs. post) by attempt within a trial. Effect sizes (partial eta squared, $\eta_{\text{p}}^{2}$) were reported in order to compliment the ANOVA *P* value for the main and interaction effects, and interpreted as small (<0.06), moderate (0.06–0.14) and large (>0.14) (46).

## Results

### Cerebrovascular reactivity to CO_2_

There was no time by trial interaction effect for resting MCAv (*P* = 0.57, $\eta_{p}^{2}$ = 0.02), MAP (*P* = 0.40, $\eta_{p}^{2}$ = 0.05) or P_ET_CO_2_ (*P* = 0.08, $\eta_{p}^{2}$ = 0.17). Accordingly, neither resting cerebrovascular resistance index (MAP/MCAv; *P* = 0.53, $\eta_{p}^{2}$ = 0.03), nor cerebrovascular conductance (MCAv/MAP); *P* = 0.63, $\eta_{p}^{2}$ = 0.02) were altered. P_ET_CO_2_ increased by 6 ± 3 mmHg after each breath hold attempt, and decreased by 10 ± 4 mmHg after each hyperventilation challenge. These values were never altered by the heading protocol (*P* = 0.49, $\eta_{\text{p}}^{2}$ = 0.05, and *P* = 0.93, $\eta_{p}^{2}$ = 0.01, respectively). MAP increased by 6 ± 7 mmHg after each breath hold attempt, and decreased by 7 ± 6 mmHg after each hyperventilation challenge. These values were never altered by the heading protocol (*P* = 0.25, $\eta_{p}^{2}$ = 0.11, and *P* = m0.97, $\eta_{p}^{2}$ = 0.01, respectively).

There was no significant time (pre vs. post intervention) by attempt interaction effect within a trial for percentage change in MCAv following each breath hold (*P* = 0.48, $\eta_{p}^{2}$ = 0.05) or hyperventilation (*P* = 0.47, $\eta_{p}^{2}$ = 0.06) attempt (Table 2). Similarly, when expressed as the change in MCAv per unit change in P_ET_CO_2_, cerebrovascular reactivity to each breath hold (*P* = 0.19, $\eta_{p}^{2}$ = 0.09) and hyperventilation attempt (*P* = 0.33, $\eta_{p}^{2}$ = 0.07) remained unaltered.

**Table 2:** Percentage increase in MCAv following the breath hold or hyperventilation challenge (*P* = 0.47 and 0.48 for the ANOVA interaction effect, respectively).

	**Percentage change in middle cerebral artery blood velocity**				
	**Attempt 1**	**Attempt 2**	**Attempt 3**	**Attempt 4**	**Attempt 5**
**Breath holding**					
Control pre	44.6 ± 10.3	42.7 ± 11.4	42.6 ± 16.0	44.5 ± 15.2	44.4 ± 14.7
Control post	39.0 ± 11.7	43.3 ± 12.9	39.6 ± 13.1	42.8 ± 15.1	41.8 ± 15.1
Heading pre	41.4 ± 13.5	36.6 ± 12.1	40.1 ± 15.8	38.9 ± 13.3	39.3 ± 12.5
Heading post	35.2 ± 12.7	40.3 ± 11.8	41.3 ± 13.9	38.5 ± 17.4	39.3 ± 14.8
**Hyperventilation**					
Control pre	−33.6 ± 8.9	−34.0 ± 7.5	−30.7 ± 8.7	−31.5 ± 7.8	−31.3 ± 9.6
Control post	−36.4 ± 13.2	−33.3 ± 8.5	−31.8 ± 10.4	−29.3 ± 10.3	−29.1 ± 8.2
Heading pre	−33.8 ± 9.0	−32.5 ± 11.4	−28.6 ± 9.9	−29.4 ± 11.1	−29.9 ± 10.3
Heading post	−32.6 ± 11.8	−30.7 ± 12.2	−29.2 ± 8.7	−25.6 ± 9.5	−25.9 ± 8.5

Data are presented as the mean ± standard deviation. *N* = 19.

### Dynamic cerebral autoregulation

**Figure 1:**
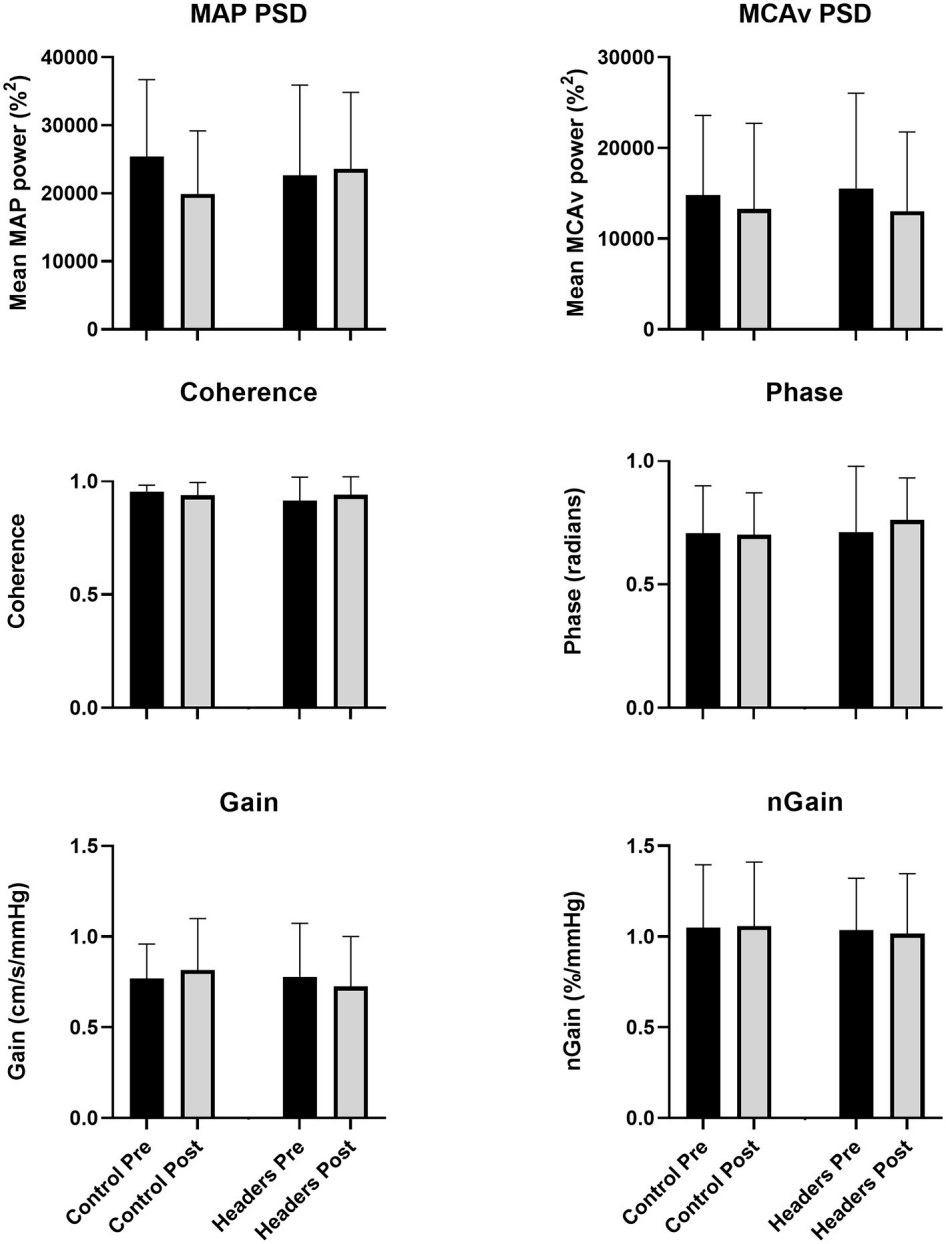
No changes in any metric of dynamic cerebral autoregulation were observed in the study (*P* > 0.14 for the ANOVA time by trial interaction effect across all outcomes). Error bars describe the standard deviation. *N* = 15 due to equipment failure in in 4 participants. MAP, mean arterial pressure; MCAv, blood velocity in the middle cerebral artery; PSD, power spectrum density; nGain, normalized gain.

Mean coherence across trials was >0.93 at all time points, indicating excellent linearity between input (MAP) and output (MCAv). There was no significant trial by time interaction effect for the mean power spectrum densities for MAP (*P* = 0.19, $\eta_{p}^{2}$ = 0.12) or MCAv (*P* = 0.66, $\eta_{p}^{2}$ = 0.01). No significant trial by time interaction effect was present for phase (*P* = 0.33, $\eta_{p}^{2}$ = 0.07), gain (*P* = 0.12, $\eta_{p}^{2}$ = 0.16) or normalized gain (*P* = 0.27, $\eta_{p}^{2}$ = 0.09) (Figure 1).

### Neurovascular coupling

**Figure 2:**
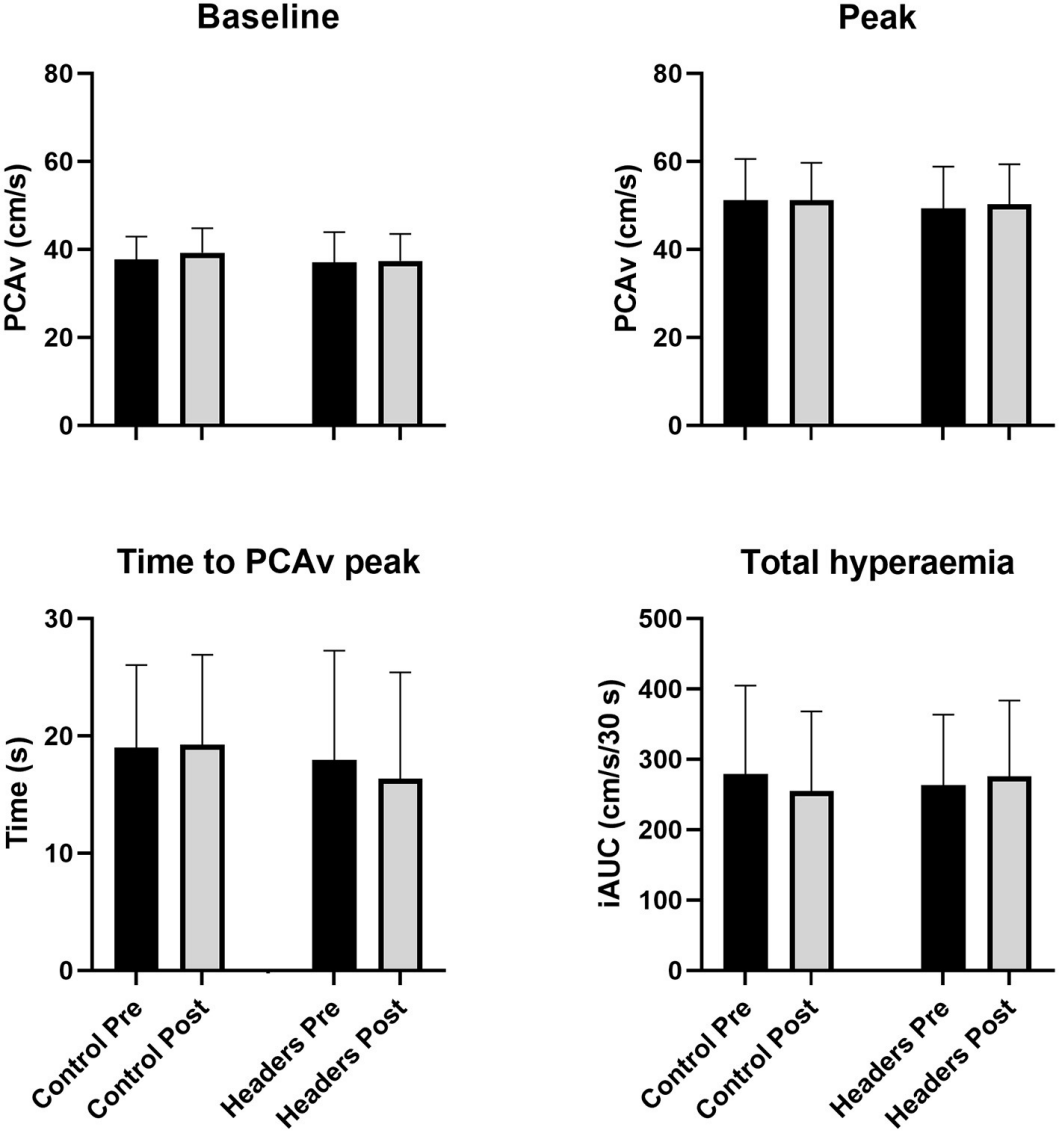
No changes in neurovascular coupling metrics were observed (*P* > 0.16 for the ANOVA time by trial interaction effect across all outcomes). Error bars describe the standard deviation. *N* = 15. PCAv, cerebral blood velocity in the posterior cerebral artery; iAUC, incremental area under the hyperaemic curve vs. time.

No significant trial by time interaction effect was apparent for baseline PCAv (*P* = 0.32, $\eta_{p}^{2}$ = 0.07). No changes in any NVC metric were observed (Figure 2). Specifically, there was no significant trial by time interaction effect for mean PCAv during the visual task (*P*=0.94, $\eta_{p}^{2}$ < 0.01), peak PCAv (*P* = 0.56, $\eta_{p}^{2}$ = 0.03), percentage increase in PCAv (*P* = 0.58, $\eta_{p}^{2}$ = 0.02), time to peak PCAv (*P* = 0.59, $\eta_{p}^{2}$ = 0.02) or total hyperaemic response (*P* = 0.14, $\eta_{p}^{2}$ = 0.15).

## Discussion

This is the first study to assess the acute effect of heading across a comprehensive range of regulatory challenges to cerebral blood flow in female footballers. Our data demonstrate that cerebrovascular reactivity to CO_2_, dCA, and NVC were unaltered after performing six purposeful headers in 1 h.

Whilst we did not observe changes in any assessment of cerebral blood flow regulation in this study, other interventional heading studies have demonstrated acute alterations in dCA (phase) (8) and NVC (9) in men. A key difference between these studies and our investigation is that they included 40 purposeful headers in a 20 min period, compared to our six headers in 1 h. Our protocol is thought to reflect the upper limit of heading exposure within a single training session or match in female footballers (14, 16, 17). In contrast, performing 40 headers is likely to exaggerate any alterations in cerebral blood flow regulation. However, despite this lack of ecological validity, such studies afford an opportunity to understand how the accumulation of heading impacts over the course of a playing career might be linked to chronic degenerative disease. Whether or not a true dose-response relationship exists between the number of headers performed and any acute alterations in cerebral blood flow regulation, or whether there is an exposure threshold for acute changes, is currently unknown. The importance of any recovery time between headers is also unknown; for example, we cannot rule out that 6 headers performed consecutively might have altered our findings. These questions remains an important area of study, especially as recent observational data indicate that a threshold effect may exist for headers accrued over a year and changes in white matter microstructure and cognitive function (4).

This difference in the number of headers prescribed clouds our ability to compare across studies, even though the nature of the heading stimulus and the methodological approach to quantifying dCA and NVC were replicated. It is worth noting that Smirl, et al. (9) observed changes in the MCAv response to the visual search task following their heading protocol, but not PCAv. Additionally, these authors did not observe alterations in PCAv during the same visual search task after one season of contact sport in men (47). Given that the PCAv response is known to be altered in the days following a concussive event (45), and perfuses the area of the brain which might be associated with contrecoup injury, it appears that heading and subconcussive impacts might not influence NVC in the posterior cerebral circulation. However, we did not simultaneously insonate the middle and posterior cerebral arteries during our cerebrovascular assessments, so we are unable to comment on any regional differences in cerebral blood flow regulation.

The data presented here should be considered in the context of the wider evidence base and the growing concern regarding the accumulation of subconcussive head impacts in sport. Cross-sectional data are available which demonstrate that cerebral blood flow regulation is altered in male footballers (7), rugby players (48) and retired boxers (49), whilst changes in dCA are observed following a season of contact sports—including football (41). Importantly, overt concussions may not be a pre-requisite for these alterations, but rather the accumulation of subconcussive impacts (7, 41, 49). Emerging evidence in adolescent women footballers indicate that the accumulation of head impacts over a season is associated with alterations in the cerebrovascular reactivity to CO_2_ (25, 50). This is a concern, given that the frequency of headers would likely increase at collegiate level (17). Therefore, we caution that the lack of observed changes in cerebral blood flow regulation in our study does not absolve the act of heading with regards to potential future neurodegenerative disease risk; i.e., the absence of evidence is not evidence of absence. Consideration needs to be given to the fact that amateur players may accumulate several thousands of headers over their years of playing the game (4).

Further work is now needed to determine whether indices of cerebral blood flow regulation and other putatively important markers implicated in the pathogenesis of neurodegenerative disease are altered across a season in adult female footballers. For example, our understanding that footballers present with impaired cerebrovascular reactivity to CO_2_ (7), and that a season of contact sports can alter dCA (41) is driven solely by data in males. Studies which are able to also quantify the headers performed, or secure head acceleration data during training, matches or across a season, preferably alongside biomarkers of axonal injury, are warranted.

The strengths of this study include the assessment of a range of regulatory challenges, the adoption of an ecologically valid heading protocol, and the recruitment of female players who might be at an increased vulnerability to deleterious change post headers (22, 24). However, our data should be considered amongst a number of limitations. Firstly, in addition to potential differences between the MCAv and PCAv responses during the visual search task, regional differences in the cerebrovascular reactivity to CO_2_ (51, 52) have been observed elsewhere. We cannot extrapolate our data beyond the single intracranial artery insonated. However, recent evidence indicate no regional differences in CO_2_ reactivity or dCA exist in fit young women (53).

Secondly, transcranial Doppler sonography only provides a valid surrogate measure of cerebral blood flow if diameter remains unchanged (54). However, this approach provides excellent temporal resolution which aids the assessment of CO_2_ reactivity (34, 55), whilst P_ET_CO_2_ has been demonstrated to be unaltered during the visual search task (9, 42) and squat stand maneuvers (8, 36) utilized in this study.

We did not control for the menstrual cycle in our study, which remains an area of debate (56, 57). However, data are available which indicate that the menstrual cycle does not influence the MCAv response to hypercapnia (58) or dCA (59), and any effect on NVC has been recently questioned (60).

Finally, it was beyond the scope of this study to quantify the magnitude of the head accelerations experienced by the players. We also did not measure blood markers of neuronal or axonal damage which might provide further insight regarding any link between repetitive heading and future neurodegenerative risk. For example, an increase in neurofilament light chain protein has recently been observed in the immediate aftermath of a heading protocol in men (61) and in males and females combined (62). Given the greater rotational acceleration experienced by women when heading (22, 23), which is associated with axonal injury (63), there is a pertinent need to consider whether this is altered by sex, or neck strength (64).

## Conclusion

There is growing concern regarding the potential link between heading over the course of a football career and future risk of degenerative disease. This study explored whether exposure to a realistic number of headers alters putatively important indices of cerebral blood flow regulation in women. We observed no acute changes in cerebrovascular reactivity to CO_2_, dCA, or NVC following six headers in 1 h in female footballers. Further study is needed to understand how these outcomes alongside other indicators of microtrauma, may be altered following a season or career in female players.

## Data availability statement

The data supporting the conclusions of this article can be made available upon reasonable request.

## Ethics statement

The studies involving human participants were reviewed and approved by University of Exeter Ethics Committee. The patients/participants provided their written informed consent to participate in this study.

## Author contributions

BB and GW designed the study. JJ, AW, OS, FB, and KL completed data collection. BB drafted the initial manuscript. All authors contributed to data analysis, interpretation, commented on subsequent revisions, and reviewed and approved the final submission.

## Funding

This work was funded by the Union of European Football Associations (UEFA) Research Grant Programme.

## Conflict of interest

The authors declare that the research was conducted in the absence of any commercial or financial relationships that could be construed as a potential conflict of interest.

## Publisher's note

All claims expressed in this article are solely those of the authors and do not necessarily represent those of their affiliated organizations, or those of the publisher, the editors and the reviewers. Any product that may be evaluated in this article, or claim that may be made by its manufacturer, is not guaranteed or endorsed by the publisher.
